# Anston attentional network for structured data based stroke risk prediction in smart aging

**DOI:** 10.1038/s41598-025-18758-5

**Published:** 2025-10-07

**Authors:** Feng Zhou, Shijing Hu, Xiaozheng Du, Zhihui Lu

**Affiliations:** 1 School of Artificial Intelligence, Shanghai Normal University Tianhua College, No. 1661 Shengxin North Road, Shanghai, 201815 China; 2 School of Computer Science, Fudan University, Shanghai, 200438 China

**Keywords:** Deep learning, Attention mechanism network, Structured data classification, Disease risk prediction, Big health, Computer science, Information technology

## Abstract

To reduce the pressure on public health services caused by the aging population, nursing homes need to predict disease risks for the elderly periodically. To improve the disease risks predicting ability of nursing homes, we designed Anston (An Attention Mechanism Network Model for Structured Data Classification) in the application scenario of innovative elderly care. The Anston model can use the physiological indicators and pathogenic factors easily collected by nursing homes to predict disease risks. In the study of disease risk prediction based on physiological indicators and pathogenic factors for thoughtful elderly care, we designed a data enhancement method, a feature weight automatic update method, and a multi-layer perceptron neural network to solve the problems of sample shortage, inconsistent feature weights, and sample imbalance. At the same time, we designed an attention mechanism network model for structured data classification based on the multi-layer perceptron neural network Developed in this paper. To fit the application scenario of competent elderly care, we propose a disease risk prediction model, Anston, based on the data enhancement method, feature automatic update method, and structured data classification attention mechanism network designed in this paper. We use public data sets and subject data as sample data in the experiment. The experimental results show that the Anston model has an accuracy of 95%, a precision of 92%, a recall of 91%, a specificity of 93%, an F1 score of 91%, and an AUC of 93% in predicting disease risks in the experiment, which have achieved the SOTA result.

## Introduction

The World Health Organization’s response to the theme of aging on its website home page reads^1^: “Healthy ageing is defined as developing and maintaining the functional ability that enables well-being in older age.” In a fact sheet on aging and health^2^: “Older people also contribute in many ways to their families and communities. Yet the extent of these opportunities and contributions depends heavily on one factor: health.” Therefore, in the application context of thoughtful elderly care, it is imperative to predict disease risks. This paper found in the research that in the application scenario for intelligent elderly care disease risk prediction, the physiological indicator values that elderly care institutions can easily collect are body temperature, blood pressure, heart rate, blood oxygen, uric acid, cholesterol, blood sugar, HDL and LDL and triglycerides. Currently, there is a lack of doctors in nursing homes^3,4^.

As for the disease factors, only Boolean values of preset disease factors can be collected, such as whether there is high blood pressure, whether there is heart disease, and whether there is a smoking habit. Therefore, the method used by hospitals to comprehensively test multiple physiological indicators of patients and guide patients to express disease factors (such as clinical symptoms) is not suitable for elderly care institutions to predict disease risks for the elderly in intelligent elderly care application scenarios.

From a health management perspective, identifying the elderly’s disease risks as early as possible can make it easier for them to obtain the best treatment period, which not only improves the health of the elderly but also reduces the pressure on public medical services. The Lancet Neurology Commission described in “Pragmatic solutions to reduce the global burden of stroke: a World Stroke Organization–Lancet Neurology Commission” that by 2050, the number of people dying from stroke worldwide each year is expected to reach 9.7 million^5^. Stroke is an acute cerebrovascular disease with high morbidity, mortality, and recurrence rates and can cause language disorders, consciousness disorders, and movement disorders^6^. When doctors in the hospital judge a patient’s risk of stroke, they will measure the patient’s height, weight, and blood pressure, as well as check the primary conditions of the patient’s heart, lungs, liver, spleen, and other vital organs^7,8^. They will also perform cerebral angiography, Head and neck magnetic resonance angiography, high-resolution magnetic resonance imaging, carotid artery B-mode ultrasound, transcranial Doppler ultrasound, and other examinations that can ultimately confirm the patient’s risk of stroke.

**Fig. 1:**
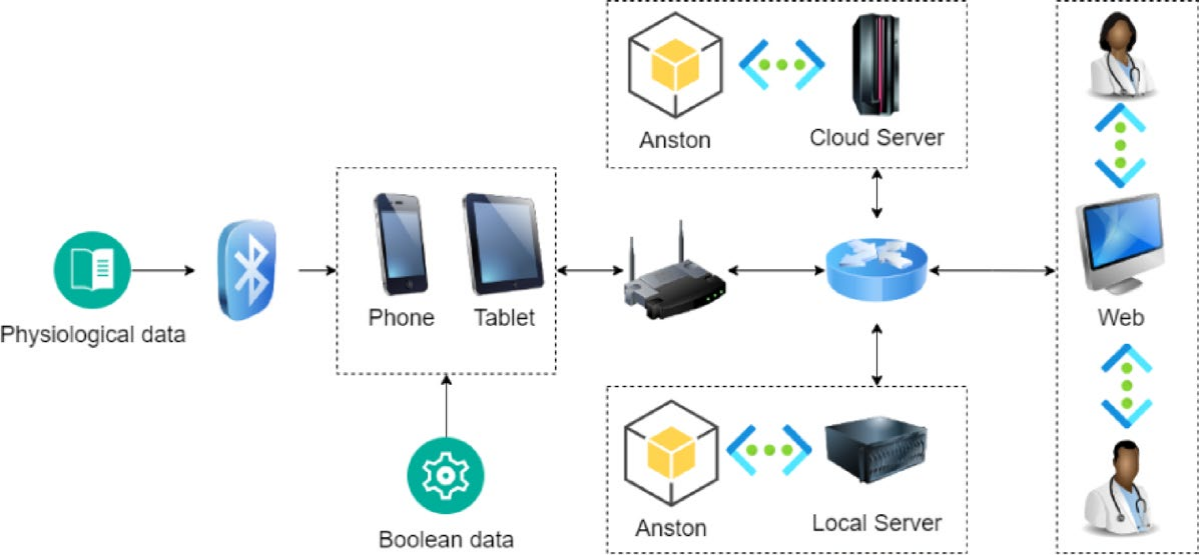
Anston model application scenarios.

Nursing homes lack medical testing equipment, and there are some common clinical factors for stroke, such as high blood pressure, heart disease, bad living habits (such as smoking), overweight, and obesity. Therefore, under the limited hardware conditions of nursing homes, the risk of stroke can be predicted by analyzing the elderly’s physiological indicators and disease factors. In response to the above description, this paper designed a disease risk prediction model called Anston (An Attention Mechanism Network Model for Structured Data Classification) when conducting disease risk prediction research based on physiological indicators and disease factors. The characteristic variables of the sample data used in Anston model training are body temperature, blood pressure, heart rate, blood oxygen, uric acid, cholesterol, blood sugar, high-density lipoprotein, low-density lipoprotein, triglycerides, and BMI that nursing homes can easily collect. The morbidity factor characteristic variable is the Boolean value of the specified morbidity factor that the nursing home institution can collect. The application scenario of the Anston model is shown in Fig. 1.

As shown in Fig. 1, the Anston model will be deployed on the local server and cloud server of the nursing home, and the local server and cloud server are hot backups for each other. Physiological indicator data are transmitted to the mobile terminal via Bluetooth, and the disease factor data are entered locally on the mobile terminal. The Anston model empowers nursing homes with disease risk prediction capabilities through API interfaces. After receiving the physiological indicators and disease factors, the Anston model deployed on the cloud server will perform relevant analysis and return the analysis results in the API interface. If the response of the Anston model deployed on the cloud server times out, the Anston model deployed on the local server will return the analysis results in the API interface.

The research goal of this paper is to predict disease risk by using body temperature, blood pressure, heart rate, blood oxygen, uric acid, cholesterol, blood sugar, high-density lipoprotein, low-density lipoprotein, triglycerides, BMI index, and disease factors. In research on disease risk prediction based on physiological indicators and disease factors, the number of samples in the current public data sets needs to be more balanced. In addition, in the actual application scenario of innovative elderly care, most new samples are those with disease risks. This sample imbalance will make the model unable to generalize. Therefore, it is urgent to solve the problem of instability in model performance due to sample imbalance. The main work of this paper on predicting disease risk is as follows:

- Given the insufficient sample data of physiological indicators and pathogenic factors, this work designed a data enhancement method.
- Because of the inconsistency of the weight value of each feature affecting stroke risk to the prediction result, which will affect the training efficiency of the model, this work integrates the loss function in the target detection network into the ensemble learning algorithm, redefines the threshold of relevant judgment in the ensemble learning algorithm, and designs a feature automatic update method based on the improved ensemble learning algorithm and machine learning interpretable framework.
- Given the problem that the model performance is unstable due to the imbalance of the number of data set samples, this work combines the automatic search optimizer to design a multi-layer perceptron neural network. It combines the perceptron neural network to innovatively design an attention mechanism network model for structured data classification.
- Given the application scenario of stroke risk prediction based on physiological indicators and pathogenic factors, this work designs a stroke risk prediction model that integrates the data enhancement method designed in this paper, the feature automatic update method, and the attention mechanism network for structured data classification.

This paper consists of six sections. The first section is an introduction, which mainly describes the research background of this paper. Section “Related work” is related work, primarily describing the current research status of this paper. Section “ Model design and implementation” is model design and implementation, primarily describing the process of Anston model design and implementation. Section “Experimental results and analysis” analyzes experimental results, mainly representing the experimental results and analysis of the Anston model. Section “Conclusion” is the conclusion, which describes primarily the results of this research and the prospects for this research.

## Related work

Many studies are currently devoted to predicting disease and stroke risks based on machine learning technology. Machine learning has also achieved good results in predicting disease risk and has produced many clinical applications. Among these studies, many studies predict disease risk and stroke risk based on physiological indicators and pathogenic factors. For example, Y. Jin et al. developed a prediction method for searching for indicators related to acute exacerbation based on random forests to improve the effectiveness of home remote health monitoring for COPD intervention^9^. The method achieved an accuracy of 75% in the experiment. W. Chang et al. used height, weight, heart rate, limb blood pressure, 24-hour dynamic blood pressure, inflammatory factors, echocardiogram indicators, white blood cell and red blood cell counts, hemoglobin (HB), urine routine indicators, blood biochemical indicators, thyroid function indicators, and urine protein indicators to propose a model based on XGBoost and SVM to predict whether hypertensive patients will develop hypertensive heart disease within three years^10^. This model achieved an accuracy of 91.7% in the experiment. Z. Huang et al. used heterogeneous electronic health records and proposed a method for predicting acute coronary syndrome based on multi-task and adversarial learning^11^. This method achieved an accuracy of 78.3%, a precision of 80.9%, a recall of 92.4%, an F1 score of 87.4%, and an AUC of 60.8% in the experiment. P. Zhang et al. proposed a model that uses sentence similarity to perform symptom similarity analysis to achieve primary disease prediction^12^. This model uses word embedding and convolutional neural networks to extract patient symptoms. This model’s F1 score and accuracy in the experiment reached 83.9%.

Tuomas Kiviniemi et al. found that patients undergoing aortic valve replacement have a high risk of stroke^13^. Brian F. Gage pointed out that anticoagulation treatment of atrial fibrillation can prevent about 64% of strokes^14^. Jae-woo Lee et al. used health examination data including age, BMI, cholesterol, hypertension, diabetes, smoking status and intensity, physical activity, drinking, history (hypertension, coronary heart disease) and family history (stroke, coronary heart disease), and used the Cox proportional hazard regression model to develop a model to predict the prevalence of stroke within ten years^15^. The AUC values ​​of this model in the experiment of the external validation data set were 0.83 for males and 0.82 for females. Ettore Beghi et al. used demographic information and clinical factors and used SAS statistical analysis tools to conclude that stroke patients are very likely to fall, fall repeatedly, and fall in a short period^16^. Among them, stroke patients with poor balance confidence have the highest risk of falling. Soumyabrata Dev et al. used age, heart disease, average blood sugar level, and hypertension to construct models for predicting stroke risk using neural networks (NN), decision trees (DT), random forests (RF), and convolutional neural networks (CNN)^17^. In the experiment, the prediction models constructed by neural networks (NN), decision trees (DT), random forests (RF), and convolutional neural networks (CNN) achieved accuracies of 77%, 74%, 74%, and 74%, respectively. Elias Dritsas et al. used a data set that included age, gender, hypertension, heart disease, and average blood sugar and proposed a stroke risk prediction method based on stacking generalization^18^. The method achieved an AUC of 98.9%, an accuracy of 98%, and a precision and recall of 97.4% in the experiment.

To achieve an automatic preliminary diagnosis of patients’ clinical narratives^19^, Y. Yu et al. extracted the chief complaint, current medical history, and past medical history from the intensive care medicine public dataset MIMIC-III. They proposed a preliminary diagnosis prediction model using an attention-based bidirectional long short-term memory (BiLSTM) network. The average accuracy of this model in the experiment reached 51.22%. C. Mugisha et al. used the intensive care medicine MIMIC-III dataset to predict pneumonia risk using structured and unstructured data in electronic health records (EHRs)^20^. They proposed an integrated model based on Adaboost and BioBERT. The AUC of this integrated model reached 87%, the F1 score reached 79%, and the average Matthews correlation coefficient (MCC) score was 89.4% in the experiment. To achieve the prediction of patient’s health status using patients’ historical electronic medical record (EMR) data^21^, S. Wang et al. used the MIMIC-III dataset. They proposed a method based on convolutional neural networks to learn robust representations using the structural and temporal information of EMR data. The AUC of this method in the experiment reached 90.06%. P. -Y. Liang et al. proposed a prediction method based on random forests to remind myocardial infarction patients to strengthen personal self-care and avoid the transition to heart failure^22^. The method achieved an accuracy of 80%, a precision of 77.1%, and an F1 score of 82.1% in the experiment. T. Wang et al. proposed a prediction method based on long short-term memory neural network to assess the risk of multiple diseases in patients in the future according to the patient’s medical records^23^. The technique achieved a Hamming score of 99.22%, a recall of 99.45%, a precision of 99.7%, an F1 score of 99.15%, and a Hamming LOSS of 0.34% in the experiment of the MIMIC dataset. N. D. T. Tran et al. proposed a health analysis system based on autoencoders^24^, few-shot learning, and multi-task learning to identify and predict COVID-19 coronavirus disease using serum antibody test results of blood samples. H. Song et al. proposed a prediction system based on a multilayer perceptron neural network to predict the incidence of diabetes after the user inputs relevant data^25^. The AUC of this system in the experiment reached 91.5%.

Z. Sun et al. proposed a new variational Hawkes process model using the data of heart failure and sepsis patients in the MIMIC-III database and the data set of heart failure patients with multiple hospitalizations to predict the patient’s future status based on the clinical observation data of the patient’s historical disease^26^. The mean square error (MSE), mean absolute error (MAE), and dynamic time wrapping (DTW) of this model in the experiment reached 2.72%, 6.31%, and 82.35%, respectively. Y. Obeidat et al. proposed a prediction method for insulin amount based on a neural network using a data set including age, gender, weight, height, diabetes period, time blood sugar, and other features to predict the appropriate amount of insulin according to the patient’s blood sugar level^27^. The mean square error of this method in the experiment was 5.79, and the average accuracy was 98.7%. To achieve primary prevention of cardiovascular disease^28^, R. Poulain et al. used an electronic health record (EHR) dataset including systolic blood pressure (SBP) and diastolic blood pressure (DBP), glycated hemoglobin (HB), blood glucose (GLC), fasting blood glucose (FBG), total cholesterol (TCL), high-density lipoprotein (good) cholesterol (HDL), low-density lipoprotein (bad) cholesterol (LDL), non-high-density lipoprotein cholesterol (NHC), triglycerides (TGC) and body mass index (BMI). They proposed a multi-target regression model based on Transformer. The average MAE of this model in the experiment reached 12.6%. M. N. I. Suvon et al. used the patient’s electronic health record (EHR) to predict mortality in patients with pulmonary hypertension^29^. They proposed a multimodal learning method based on an attention mechanism and weighted summation to predict the one-year mortality rate of patients with pulmonary hypertension. The AUC score of this method in the experiment reached 89%. Y. Meng et al. used the Transformer network model to perform bidirectional representation learning on electronic health record data and proposed a deep learning model for predicting future depression time^30^. The area under the operating characteristic curve (AUC) and area under the precision-recall curve (AUPR) of this model in the experiment reached 85% and 76%, respectively. To improve the accuracy of patient disease prediction, F. Yu et al. used the EHR data of patients with circulatory system diseases and heart failure in the open-source dataset MIMIC^31^. They proposed a disease risk prediction method based on a logical perception network. The accuracy of this method in the experiment was 91.38%, and the F1 score was 59.22%. S. Nandy et al. used a Brazilian COVID-19 patient dataset, including throat discomfort, fever, dyspnea, headache, and gender^32^. They proposed a framework for remote health monitoring and disease prediction based on neural networks. This framework achieved an accuracy of 99.8% in the experiment.

Tianyu Liu et al. used an unbalanced medical dataset, including average glucose, hypertension, heart disease, BMI, and other features, and designed a stroke prediction method based on a DNN model^33^. The false negative rate obtained by this method in the experiment was 19.1%, and the accuracy was 71.6%. Stephen Bacchi et al. extracted information about stroke patients’ age, gender, time from stroke onset to visit, National Institutes of Health Stroke Scale (NIHSS) at visit, blood pressure at visit, etc., from the existing databases of two tertiary hospitals in South Australia, and proposed a method to predict the outcome of thrombolytic therapy for stroke patients based on convolutional neural networks (CNN) and artificial neural networks (ANN)^34^. The accuracy of this method in the experiment was 71%, and the F1 score was 74%. Ching-Heng Lin et al. used the collected data from stroke patients, including clinical care during hospitalization, in-hospital complications, and stroke risk factors^35^. They proposed a stroke prediction method based on SVM, random forest, artificial neural network (ANN), and hybrid artificial neural network (HANN) using 10-fold cross-validation. The AUC value of this method in the experiment reached 94%, and the precision, recall, and F1 scores all went more than 85%. Sheng-Feng Sung et al. used clinical feature data of emergency department triage, including chief complaint, triage level, hypertension, diabetes, hyperlipidemia, heart disease, and previous stroke, to develop a stroke alarm trigger based on logistic regression and random forest to support clinical decision-making during emergency triage^36^. Yosuke Hayashi et al. used stroke prediction data in the prehospital environment, including variables such as symptoms, physiological data, and medical history, and proposed a prehospital stroke diagnosis algorithm based on XGBoost and SHAP^37^. The AUC obtained by this algorithm in the experiment was 98%.

To predict the recurrence of stroke patients^38^, H. Xu et al. used 205 cases in the hospital and 2954 cases from the screening cohort (2015–2017), including gender, age, anticoagulants, hypertension, diabetes, smoking, drinking, body mass index (BMI), and low-density lipoprotein (LDL), and applied instance filters and weight-based Transfer learning methods to Develop a stroke risk prediction model. The best PR-AUC of this model in the experiment reached 75.8%. J. Chen et al. used external stroke data (such as essential health records, blood tests, etc.) and chronic disease data and proposed a stroke risk prediction method based on a hybrid deep transfer learning framework^39^. The accuracy of this method in the experiment reached 72.9%, and the AUC value reached 78.1%. Ruixuan Huang et al. used a data set including patient demographics, diagnosis, surgery, various physiological index test results, and medications to train classifiers and models to predict the six-month mortality rate of patients with hemorrhagic and ischemic stroke^40^. The AUC of these classifiers and models in the prediction experiment of the six-month mortality rate of hemorrhagic stroke and ischemic stroke exceeded 80%. Yen-Nung Lin et al. used a data set including demographic characteristics, clinical data, diagnostic records, stroke history, and complications retrieved from medical records, calculated descriptive statistics based on the statistical analysis software SAS, and established the ability of post-acute care activity assessment to predict functional improvement in the three areas of early, mid-term, and late recovery^41^. Yauhen Statsenko et al. used a supervised machine learning model by combining comprehensive meteorological and clinical demographic data of 160 stroke patients^42^. They trained a model that can accurately predict stroke outcomes using stratified 10-fold cross-validation technology. The F1 score of this model in the experiment reached 90%, and the AUC value reached 89.6%. Seong Hwan Kim et al. used Korean Multicenter Prospective Stroke Registry data. They proposed a prediction model for early neurological deterioration in stroke based on LightGBM and the machine learning interpretable framework Shapley^43^. The AUC of this model in the experiment reached 77.8%.

**Table 1:** Research on disease risk and stroke risk prediction based on physiological indicators and pathogenic factors.

Researcher	Disease	Basic Algorithm	Accuracy
P. -Y. Liang^22^	Heart failure	Random forest	80%
F. Yu^31^	Circulatory system diseases	Logical perception network	91.38%
S. Nandy^32^	COVID-19	DNN	99.8%
Tianyu Liu^33^	Stroke	DNN	71.6%
Stephen Bacchi^34^	Thrombolytic therapy for stroke	CNN + ANN	71%
J. Chen^39^	Stroke	DNN	72.9%

From the above description and Table 1, we can see that in the field of disease risk and stroke risk prediction based on physiological indicators and pathogenic factors, the accuracy of heart failure risk prediction reached 80%, the accuracy of circulatory system disease risk prediction reached 91.38%, the accuracy of thrombolytic therapy results for stroke reached 71%, and the accuracy of stroke risk prediction reached 72.9%. In summary, the existing research on disease risk and stroke risk prediction based on physiological indicators and pathogenic factors has achieved good results, but there is still room for improvement. To fit the application scenario of intelligent elderly care stroke risk prediction, the data used in the research must be data that is easy for elderly care institutions to collect. In this paper, when conducting stroke risk prediction research, data that is easy to collect in elderly care institutions is used. In addition^44,45^, since it is a common phenomenon that the proportion of samples with stroke risk in the newly added samples in this application scenario is relatively large^46,47^, the problem that sample imbalance will bring instability to model performance is also a common problem in this application scenario. To improve the prediction effect of stroke risk and solve the common problem of unstable prediction model performance caused by imbalanced data sets in innovative elderly care applications^48–50^, this paper integrates the loss function in the target detection network into the ensemble learning algorithm. It redefines the threshold of relevant judgment in the ensemble learning algorithm. A multi-layer perceptron neural network and an attention mechanism network for structured data classification are designed.

## Model design and implementation

In this section, we describe the design and implementation process of the Anston model we proposed from the perspectives of data augmentation methods, automatic feature weight update methods, attention mechanism network models for structured data classification, model structure, sample feature preprocessing and feature classification, model algorithms, and model parameter optimization.

### Data augmentation methods

The sample data used in this paper are MIMIC-III1.4, the stroke risk prediction data set “Cerebral Stroke Prediction-Imbalanced Dataset” published on Kaggle, and subject data from a nursing home. MIMIC-Ⅲ1.4(V1.4) is a public data set of the United States Medical Information Mart for Intensive Care-Ⅲ1.4(V1.4). Since the data in MIMIC-III1.4 are all disease risk data^51^, the “Cerebral Stroke Prediction-Imbalanced Dataset” data set is imbalanced data^33^. Therefore, during data preprocessing, this paper designed a data enhancement method to supplement the sample data of physiological indicators and disease factors. Because in research on disease risk prediction, most of the new sample data are data with disease risk. In order to improve the sample data^52^, this section uses the physiological indicators and pathogenic factors that can be easily collected by nursing homes in imaginative elderly care application scenarios and designs a data enhancement method based on the standard medical numerical range in the physiological indicator knowledge base and the OEGFCM-SMOTE sampling technology proposed by Karim El Moutaouakil et al. Although physiological indicators and disease factors are text data, they are all numerical data after collection. Therefore, methods such as synonym substitution, random insertion, random deletion, random exchange, and synonym substitution used in natural language are not applicable in this application scenario. This paper takes stroke disease as an example. The primary process of the data enhancement method based on the average medical value range of physiological indicators and corresponding pathogenic factors is summarized as follows.

**Fig. 2:**
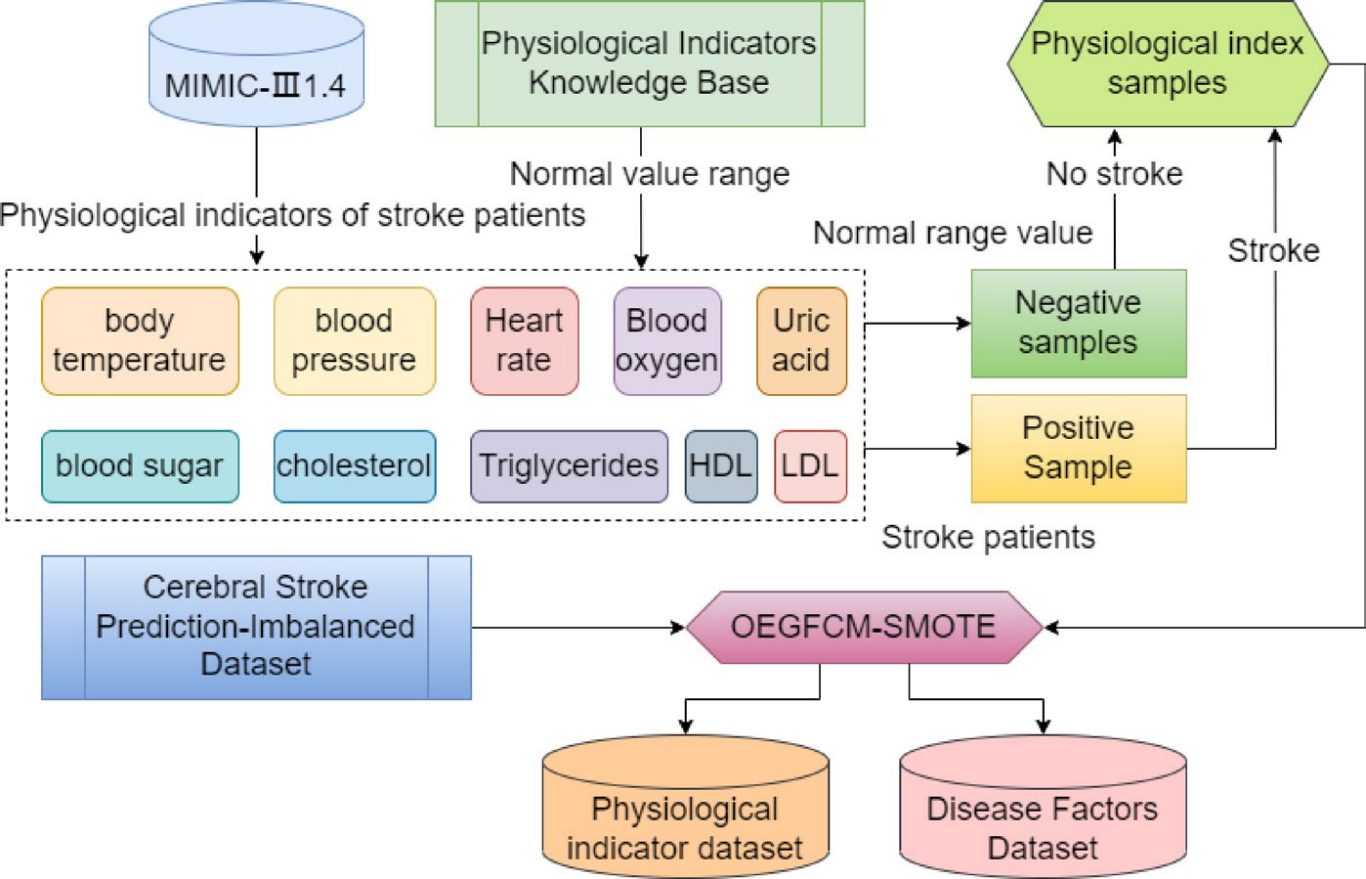
Symptom expression image feature preprocessing method flow.

The primary process of the physiological index and pathogenic factor data enhancement method in Fig. 2 is as follows:

- The body temperature, blood pressure, heart rate, blood oxygen, uric acid, cholesterol, blood sugar, high-density lipoprotein, low-density lipoprotein, and triglyceride values ​​of stroke patients are obtained from MIMIC-Ⅲ1.4 to form positive samples.
- The average value range of body temperature, blood pressure, heart rate, blood oxygen, uric acid, cholesterol, blood sugar, high-density lipoprotein, low-density lipoprotein, and triglyceride is obtained from the physiological index knowledge base, and average values ​​are randomly generated within this range to form negative samples. The above positive and negative samples are used to create physiological index samples.
- The OEGFCM-SMOTE sampling technology is used to balance the physiological index samples and the “Cerebral Stroke Prediction-Imbalanced Dataset” dataset and generate the physiological index and pathogenic factor datasets.

### Feature weight automatic update method

The LightGBM algorithm is a new lightweight boosting framework proposed by Microsoft based on the integrated learning algorithm XGBoost algorithm. This framework supports parallel learning, has significant data processing capabilities, and has the advantages of fast training speed, high training efficiency, low memory usage, and high accuracy. Therefore, this ensemble learning algorithm framework is suitable for application scenarios where feature weights are automatically updated. The imbalance in the number of positive samples and negative samples and the imbalance in the number of easy-to-classify samples and difficult-to-classify samples will cause instability in the performance of the prediction model. This paper integrates the Focal loss function in the target detection network into the ensemble learning algorithm LightGBM. It redefines the baseline threshold for classification in the LightGBM algorithm to solve this problem. In the application scenario of disease risk prediction for innovative elderly care, the number of samples in the two categories of disease risk and no disease risk is different. The number of new samples with disease risk is larger than the number of new samples without disease risk. Therefore, it is necessary to lower the classification baseline threshold below 0.5, that is, to make the model tend to believe that the newly added samples have a higher probability of being free of disease risk. Through multiple experimental, iterative tests, this paper finally redefined the classification benchmark threshold as 0.327. The new classification benchmark in the ensemble learning algorithm is shown in formula (1).

1 $$\:\mathcal{y}=\left\{\begin{array}{c}0,\:\:\:\:\:\:\:{\mathcal{p}}_{\mathcal{y}}<0.327\\\:1,\:\:\:\:\:\:\:{\mathcal{p}}_{\mathcal{y}}\ge\:0.327\end{array}\right.$$

**Fig. 3:**
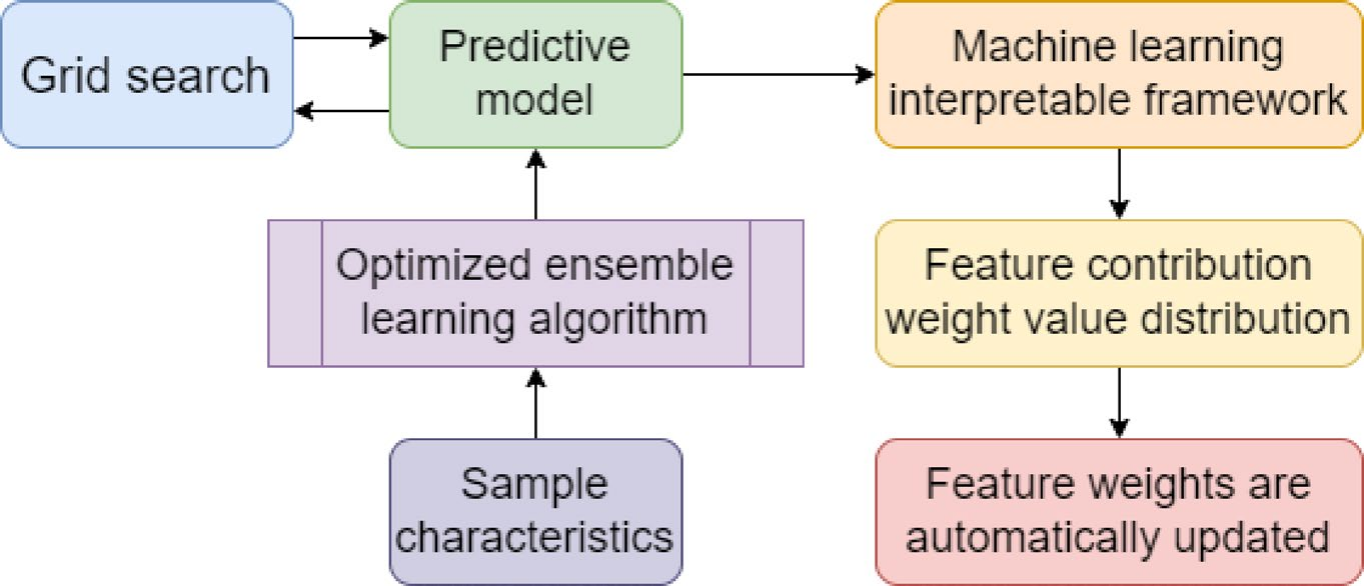
Feature weight automatic update method process.

Since there are many types of diseases involved in the application scenario of disease risk prediction for innovative elderly care, the contribution weight of each characteristic that affects disease risk to the model prediction results is sometimes very different. Therefore, to further improve the versatility and robustness of the model proposed in this paper, this paper designs a method to automatically update feature weights based on the machine learning interpretable framework and optimized ensemble learning algorithm. This method uses a machine learning interpretable framework to analyze the results of the contribution of input features to model prediction results. It automatically adjusts the weight of input features to improve the versatility and robustness of the model. The specific process is shown in Fig. 3.

The specific process of the feature weight automatic update method in Fig. 3 is as follows:

- The sample features are input into the initial classification prediction model constructed by the ensemble learning algorithm designed in this paper.
- Load the machine learning interpretable framework and analyze the contribution weight values of features to the prediction results.
- Output the contribution weight value of each feature to the prediction result and update the feature weight.

### Attention mechanism network model for structured data classification

**Fig. 4:**
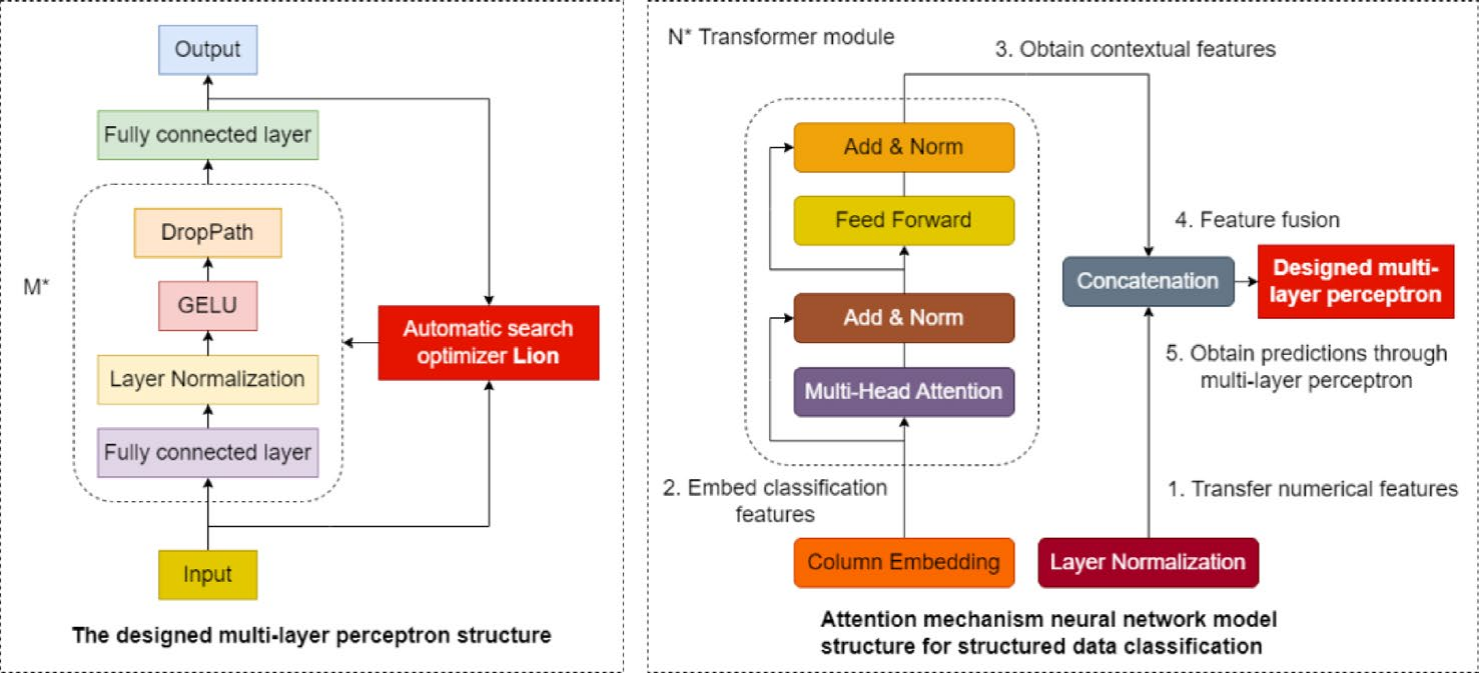
An attention mechanism network model for structured data classification.

This paper aims to improve the training efficiency, accuracy, and stability of the model in the use scenario of intelligent elderly care disease risk prediction. We first design a new multi-layer perceptron neural network. Then, referring to the TabTransformer model structure, a new attention mechanism network model for structured data classification applications was designed using the multi-layer perceptron. When designing a multi-layer perceptron, to speed up the convergence of the model and avoid the phenomenon of gradient disappearance during backpropagation, we added horizontal normalization Layer Normalization to the hidden layer. When designing multi-layer perceptron in this work, the purpose is to improve the training efficiency and prediction accuracy of the designed network model and to prevent model overfitting. Since DropPath is a random point-to-layer closure, this paper uses DropPath and the activation function GELU in the relevant module structure. In order to further improve model training efficiency and reduce memory usage, this work designs a multi-layer perceptron using the automatic search optimizer Lion proposed by Google Brain in 2023^53^. Figure 4 is the specific model structure.

Figure 4 shows the structure of an attention mechanism network model designed by this paper for structured data classification when researching disease risk prediction based on physiological indicators and disease factors. The primary process of the attention mechanism network model designed in this work for structured data classification is summarized as follows:

- Pass the standardized numerical features of structured data to the feature fusion module.
- Embed categorical features in structured data.
- The embedding passes the contextual embedding obtained N more times through the Transformer module.
- The feature fusion module cascades contextual embedding features and numerical features.
- The designed multi-layer perceptron outputs the prediction results of the model.

### Model structure

**Fig. 5:**
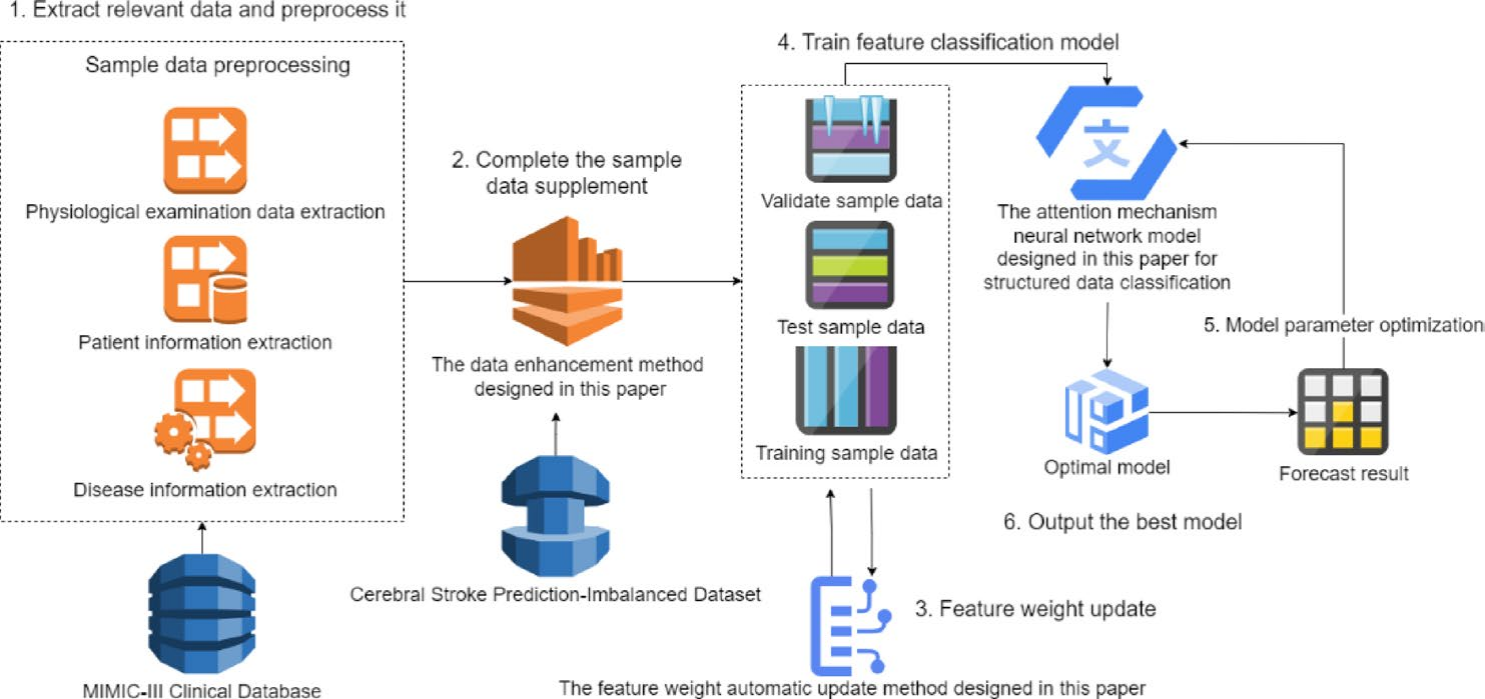
Model structure for Anston disease risk prediction.

The design of the model structure for disease risk prediction based on physiological indicators and disease factors proposed in this paper is shown in Fig. 5.

In Fig. 5, the primary process of the Anston model for disease risk prediction based on physiological indicators and disease factors is as follows:

- The disease information, patient information, and patient outpatient examination data of stroke patients are extracted from the MIMIC-III Clinical Database and preprocessed.
- Using the preprocessed MIMIC-III data and the “Cerebral Stroke Prediction-Imbalanced Dataset” data set, based on the data enhancement method designed in this paper, the sample data is improved, and the obtained data set is divided into a training set, a verification set and a test set.
- Use the feature weight automatic update method designed in this paper to analyze the prediction results, output the feature contribution value distribution, and update the sample feature weights.
- Use the attention mechanism network designed in this paper for structured data classification as the base model to train the prediction model.
- Use the Spider Wasp Optimizer (SWO) algorithm to optimize the parameters set by the model and adjust the relevant parameters of the feature classification model based on the optimization output results of the SWO algorithm^54^.
- Iterative training is performed until the model Accuracy reaches 95%, Precision reaches 92%, Recall reaches 91%, Specificity reaches 93%, F1 score reaches 91%, and Area Under the ROC Curve value reaches 93%. Finally, the best prediction model is output.

To make the constructed model suitable for real-time screening of disease risks in imaginative elderly care application scenarios, we considered the model’s expressiveness, interpretability, and implementation speed when designing it. The model composition is shown in Table 2.

**Table 2:** Model composition.

Logical blocks	Sublayers	Number of layers	Activation/regularization
Embed	Linear	1	\
Self-Attention×4	• Multi-Head Attn • Add & Norm • FFN (2 Linear) • Add & Norm	4 × 3 = 12	GELU · Dropout 0.1 · DropPath 0.2
Cross-Attention	Multi-Head Attn + FFN	3	GELU · Dropout 0.1 · DropPath 0.2
Global MLP Head	Linear → GELU → Linear → GELU → Linear	3	Dropout 0.1
Total		19	

As shown in Table 2, the proposed model comprises 19 explicit, trainable layers. Embed is to map the original input without activation and regularization uniformly and only perform linear Transformation so that the subsequent attention module uses a unified dimension. Each Block of Self-Attention consists of 1 multi-head self-attention and two layers of a feed-forward network (FFN). We stacked 4 Blocks in total, resulting in a depth of 12 layers. The activation function is GELU, and Dropout Rates of 0.1 and 0.2 are used to suppress overfitting. Cross-attention enables the network to explicitly learn the interaction between two types of features connected to two layers of the feed-forward network (FFN) rather than relying on simple splicing. The Global MLP Head consists of linear layers, featuring GELU and Dropout (0.1) in the middle two layers, which output logits and then predict results after applying softmax. When designing the hidden layer, we set the bottleneck dimension of the feed-forward network (FFN) to 4×embed, which is sufficient to express nonlinearity without introducing too many parameters. To alleviate the vanishing gradient of the deep network, the LayerNorm we use uses Pre-LN. To significantly reduce overfitting, we use DropPath to randomly skip the entire residual branch with Block, with a probability of 0.2, and combine it with Dropout.

In the design process of the Anston model, we use the improved LightGBM to update the feature weights. We utilize Layer Normalization, DropPath, GELU, the automatic search optimizer Lion, and fully connected layers to construct a multi-layer perceptron. We then use this multi-layer perceptron and refer to the TabTransformer model structure to design an attention mechanism network model structure for structured data classification. At the same time, two binary classification models (with two outputs: risky and non-risk) were trained using two datasets of physiological indicators and disease factors. Finally, the output results of the two models are combined using AND and OR operations to obtain three results: high risk, low risk, and no risk. The loss function used in the feature weight update stage is the Focal loss function, which is frequently used in target detection networks. In contrast, the loss function used in MLP-Attention is Cross-Entropy (CE).

### Sample feature preprocessing and feature classification

The sample data in this paper uses the MIMIC-III Clinical Database, the “Cerebral Stroke Prediction-Imbalanced Dataset” data set publicly available on Kaggle and related data of subjects in a nursing home. This section uses the feature weight automatic update method designed in this paper to analyze the impact of the prediction results. Based on the attention mechanism network for structured data classification designed in this paper, the feature classification prediction model is designed and trained^55,56^. The main steps for sample data preprocessing and feature classification are as follows:

- Uniformly complete rows with missing attribute values in the sample data and uniformly convert text characters into numerical characters.
- The character features and OrdinalEncoder uniformly encodes classification labels in the sample data.
- Load the feature weight automatic update method designed in this paper.
- Analyze the contribution of the prediction results and adjust the sample feature weights based on the analysis results.
- Use the attention mechanism network for structured data classification designed in this paper to define a feature classification prediction model and load the model.
- Analyze the loss value between features and prediction results, and iteratively train the feature classification prediction model until the accuracy, precision, recall, specificity, F1 score, and AUC value of the model reach the preset thresholds, respectively.

In this paper, our overall ideas and specific technical methods for sample feature preprocessing are shown in Table 3.

**Table 3:** The overall Idea and technical approach of sample feature preprocessing.

Steps	Target	Technical
Data extraction	Extract features from Dataset	Mapping, SQL JOIN
Align units with time	Ensure consistent dimensions	Map table, take median
Anomaly & missing analysis	Analyze missing patterns	Interval trimming, MAR/MCAR
Missing imputation	Stratified median imputation	IterativeImputer, logical default
Normalization/Encoding	Conform to model training	Z-score (continuous)

As shown in Table 3, during feature preprocessing, we used medical interval clipping and MICE/stratified median interpolation to make continuous features NA < 2%; Boolean features were simplified and filled with logical defaults; and normalized and input into the training network model^57^. When handling missing values, we follow the principle of first judgment, then stratification, and finally regression. For continuous features, all records identified as exceeding the medical interval, missing instrument codes, or filled with zero are marked as ‘NA.’ MICE (multiple imputations by chained equations, with body temperature, heart rate, blood pressure, etc., as predictors) is used to fill in the remaining gaps iteratively. If zero itself for Boolean or categorical features indicates that there is no risk factor, the entries that are indeed missing information are set with Unknown dummy variables for model recognition.

**Fig. 6:**
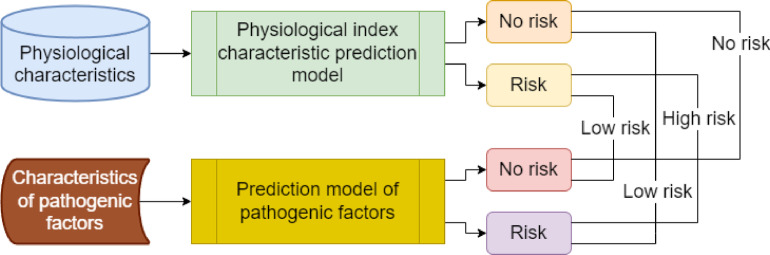
Prediction model output logic.

The output logic of the prediction model for feature classification of physiological indicator characteristics and pathogenic factor characteristics is shown in Fig. 6.

As shown in Fig. 6, the prediction model finally outputs three classification results: no risk, low risk, and high risk. When the classification result of the physiological indicator feature is no risk, and the classification result of the pathogenic factor feature is no risk, the prediction model finally outputs no stroke risk. When the physiological indicator feature’s classification result is risky, and the pathogenic factor feature’s classification result is risky, the prediction model finally outputs high stroke risk. When the classification result of the physiological indicator feature is no risk, and the classification result of the pathogenic factor feature is risky, the prediction model finally outputs low stroke risk. When the physiological indicator feature’s classification result is risky, and the pathogenic factor feature’s classification result is no risk, the prediction model finally outputs low stroke risk.

### Model parameter optimization and model algorithm

**Fig. 7:**
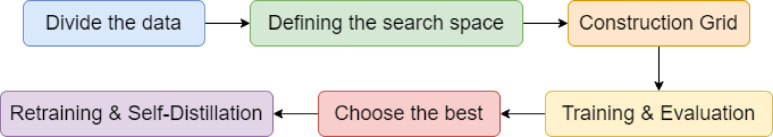
GridSearchCV Process.

Five-fold cross-validation is to divide the sample data into five parts, using four parts for training and one part for testing^4^. After completing five experiments, output the average value. When using five-fold cross-validation training sample data, this paper divides the test data into two equal parts, using one part for verification and one for testing. When implementing the feature weight automatic update method, this paper first sets a range value for the basic parameters of the prediction model, parameters that affect accuracy, parameters that affect overfitting, and parameters that affect training speed. Then, use the grid search method (GridSearchCV) to obtain the scores of relevant parameter values. Then, after iterative training, the optimal values of the relevant parameters are obtained. Finally, the feature weight automatic update method based on the design and training of the integrated learning algorithm and machine learning interpretable framework is output. The process of the grid search method (GridSearchCV) is shown in Fig. 7.

As shown in Fig. 7, to ensure that there is no overfitting during parameter optimization and to provide robust test indicators, we employ five-fold stratified cross-validation (stratified k = 5). 80% of each fold is used for the training set, and the training set and validation set are divided in a ratio of 9:1. The remaining 20% is used for hold-out testing. To complete the search within the limited 48 combinations and avoid an exponential explosion, we defined the search space in terms of four types of parameters in the optimization process: basis, affecting accuracy, controlling overfitting, and training efficiency. To improve training efficiency, we utilize ParameterGrid to generate Cartesian products when constructing the grid, resulting in 48 groups of configurations. During the training and evaluation process, LightGBM is trained for each config, and the evaluation indicators are calculated. The configuration with high evaluation indicators and training time in the top 25% is selected. To improve generalization and reduce variance, the optimal parameters are used for retraining on the training set. The adjusted parameters are shown in Table 4.

**Table 4:** The main parameters of GridSearchCV optimization.

Category	Parameter	Function	Rules
Model capacity	num_leaves∈ {32, 64, 128}	Affecting expressiveness	Until no obvious benefit
	max_depth∈ {4, 6, 8}	Prevent overfitting	> 8 noise; <4 underfitting
Learning rate	learning_rate∈ {0.05, 0.1}	Adjust convergence speed	< 0.05 converges slowly, > 0.1 oscillate
Column sampling	feature_fraction∈ {0.8, 1.0}	Improve generalization	< 0.8 loses key information
Regularization	lambda_l1∈ {0, 0.1}	Sparse leaf weights	Based on evaluation indicators
	lambda_l2∈ {0, 0.1}	Suppress large weights	Based on evaluation indicators
	min_gain_to_split∈ {0, 0.05}	Limit weak splits	Based on evaluation indicators
Accuracy	max_bin∈ {63, 255}	Histogram bin number	Based on evaluation indicators

As shown in Table 4, when we proposed the Anston model, in the process of adjusting parameters using the grid search method (GridSearchCV), we set the rules for four types of parameters: basis, influencing accuracy, controlling overfitting, and training efficiency, based on the indicator values ​​of each evaluation, the scenario requirements of competent elderly care, and literature and empirical data.

**Fig. 8:**
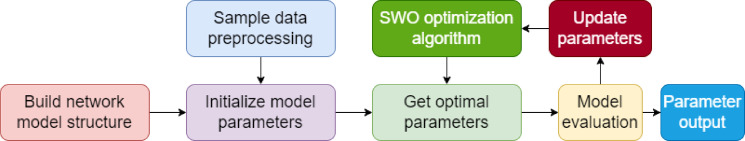
SWO Spider Bee Optimization Algorithm Parameter Optimization Process.

When optimizing the parameters of the designed attention mechanism network model for structured data classification, this paper uses the SWO spider-bee optimization algorithm with fast search speed and high solution accuracy^54^. The primary process of the SWO algorithm optimizing model parameters is shown in Fig. 8.

**Fig. 9:**
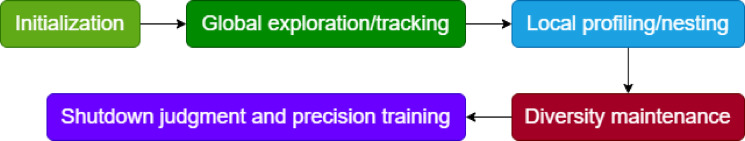
The main process of SWO spider bee optimization algorithm.

Figure 8 is the process of optimizing model parameters by the Spider Bee optimization algorithm, and finally outputs the best parameter values. The main process of the SWO optimization algorithm is shown in Fig. 9.

As shown in Fig. 9, to obtain uniform and non-repetitive samples, we use Latin Hyper-cube Sampling (LHS) for initialization; that is, in the multidimensional parameter space, each dimension is divided into several intervals, and then the sampling points are guaranteed to fall exactly once and only once in each interval in each dimension. In the global exploration/tracking process, 70% of the bees use Searching to search in large steps, and 30% of the bees use Following to converge to the current optimum. In the local analysis/nesting process, the top 10% of the bees are selected, and a single dimension is perturbed using a long-tail random step size to fine-tune the search for high-performance indicator areas. In the diversity maintenance stage^58^, each dimension inherits its parent’s characteristics with a certain probability, and only a few drones are differentially mutated to enhance the population’s activity further and eliminate the tail of 20% of the bees. In the termination and fine training stage, it is terminated when no further improvement is achieved, and the optimal bee parameters are used for complete finetuning. The algorithm of the disease risk prediction model based on physiological indicators and disease factors in this paper is summarized as shown in Algorithm 1.

**Figure:**
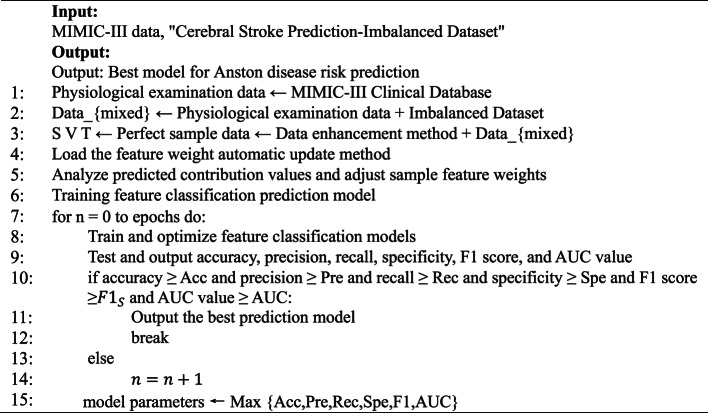
**Algorithm 1: Anston disease risk prediction model algorithm.**

In Algorithm 1, $\:S$ represents training sample data, $\:V$ represents verification sample data, $\:T$ represents test sample data, and epoch represents the number of training iterations. $\:Acc$ represents the accuracy threshold, which is preset to 95% in this paper. $\:Pre$ represents the accuracy threshold, which is preset to 92% in this paper. $\:Rec$ represents the recall rate threshold, preset to 91% in this paper. $\:Spe$ represents the specificity threshold, preset to 93% in this paper. $\:{F1}_{S}$ represents the F1 score threshold, which is preset to 91% in this paper. $\:AUC$ represents the AUC value threshold, which is preset to 93% in this paper.

To accurately predict disease risks in the smart elderly care application scenario, we integrated the automatic update of feature weights, ensemble learning robustness, and the attention mechanism of structured data into the design of the Anston model. In the data enhancement design process, we innovatively applied the OEGFCM-SMOTE sampling technology to enhance physiological indicator datasets and disease factor datasets. In the design process of automatically updating feature weights for ensemble learning robustness, we innovatively employed a dual optimization strategy for the loss function and threshold, updating the weights online by calculating SHAP in each epoch. In the design process of the structured data classification model, we integrated horizontal normalization, Layer Normalization, DropPath, the activation function GELU, an automatic search optimizer, Lion, and an embedded TabTransformer multi-head structure. When designing risk decision-making, we output three-level labels of no risk, low risk, and high risk based on clinical “two-stage screening” and interpretability rules, which not only maintain the convenience of model automation but also seamlessly connect with the traditional “screening-diagnosis-intervention” path, making it easy for elderly care institutions to implement it.

## Experimental results and analysis

In this section, we mainly describe the evaluation indicators, prediction results, feature contribution analysis methods, and model optimization used when conducting Anston model experiments. During the implementation of the Anston model, the software platforms we used were: Ubuntu Server 18.04, Python 3.7.6, Keras 2.6.0, LightGBM 3.3.2, interpret 0.3.0, Tensorflow-GPU 2.6.0, XGBoost 1.6.1, Torch 1.12.1, Numpy 1.18.5, Pandas 1.3.5, sklearn 1.1.

### Sample data and data set division

The MIMIC-III1.4 data set consists of four types of tables: term dictionary, patient information, outpatient treatment, and ICU treatment. The data in the MIMIC-III1.4 (V1.4) data set include physiological indicators, laboratory tests, imaging reports, hospitalization time, and survival data. The author of this paper has completed the “Protecting Human Research Participants” course on the NIH (National Institutes of Health) website and obtained data use rights (certificate number: 48686713).

There are up to 10,308 inspection items in the CHARTEVRNTS table in the MIMIC-III1.4 data set^40^. The items extracted in this paper are Invasive Blood Pressure systolic, Invasive Blood Pressure diastolic, Heart Rate, Skin Temperature, PAR-Oxygen saturation, Blood Glucose, Cholesterol, Uric Acid, Triglyceride, HDL, and LDL measured. There are up to 587 inspection items in the LABEVENTS table. The items extracted in this paper are Oxygen Saturation, Cholesterol Ratio (Total/HDL), Cholesterol, HDL, Cholesterol, LDL, Calculated, Cholesterol, LDL, Measured, Cholesterol, Total, Triglycerides, Uric Acid, and Triglycerides.

“Cerebral Stroke Prediction-Imbalanced Dataset” is a typical imbalanced data set collected from HealthData.gov, containing eleven features^41^, with 43,400 record samples containing 783 stroke events, accounting for only 1.18% of the whole. The attributes of this stroke prediction data set include ID, gender, age, hypertension, heart disease, ever_married, work_type, residence_type, avg_glucose_level, BMI, smoking_status, and stroke. Except for ID, gender, age, avg_glucose_level, BMI, and stroke, these attribute data are all stroke incidence factor data. The numerical types of the data are all Boolean values. These incidence factor data belong to nursing homes and can easily collect preset data.

The final sample size distribution of the physiological indicator dataset, pathogenic factor dataset, subject physiological indicator dataset, and subject pathogenic factor dataset is shown in Table 5.

**Table 5:** The number of samples in this paper’s dataset before and after enhancement.

Dataset category	Sample type	Original quantity	Used quantity after enhancement	Imbalance rate after enhancement
Physiological indicators	Negative sample	0	6000	
Physiological indicators	Positive sample	836	5000	120%
Pathogenic factors	Negative sample	42,617	6000	
Pathogenic factors	Positive sample	783	5000	120%
Physiological indicators of subjects	Negative sample	176	1200	
Physiological indicators of subjects	Positive sample	35	1000	120%
Pathogenic factors of subjects	Negative sample	150	1200	
Pathogenic factors of subjects	Positive sample	29	1000	120%

In this paper, the physiological index dataset and the disease factor dataset were divided into a training set, validation set, and test set in a ratio of 7:2:1. The subject physiological dataset and the subject disease factor dataset were all used as another test set for further testing of the prediction model performance indicators.

### Evaluation metrics

In this paper, six indicators are used to evaluate the disease risk prediction model: accuracy, precision, recall, specificity, F1 score, and AUC value.

2 $$\:Accuracy=\frac{TP+TN}{TP+TN+FP+FN}\times\:100{\%}$$

3 $$\:Precision=\frac{TP}{TP+FP}\times\:100{\%}$$

4 $$\:Recall=\frac{TP}{TP+FN}\times\:100{\%}$$

5 $$\:Specificity=\:\frac{TN}{TP+FN}\:\times\:100{\%}$$

6 $$\:F1=\frac{2TP}{2TP+FP+FN}\times\:100{\%}$$

Among them, TP represents true positives, TN represents true negatives, FP represents false positives, and FN represents false negatives, as shown in the confusion matrix in Table 6.

**Table 6:** Comparison of experimental results of different optimizers.

Sample type	Predicted as a normal sample	Predicted as an attack sample
Normal sample	TN	FP
Attack sample	FN	TP

The ROC (Receiver Operating Characteristic) curve was first used in radar signal detection to distinguish signals from noise. Later, researchers used it as an evaluation index to evaluate the prediction ability of classification models^59^. The ROC curve is proposed based on the confusion matrix. Taking the false positive rate (FPR) as the abscissa and the actual positive rate (TPR) as the ordinate, the ROC curve is drawn. AUC (Area Under ROC Curve) is the area under the ROC curve. When comparing the effects of classification models, draw the ROC curve of each model and compare the area under the curve as an indicator to judge the quality of the classification model^60^.

### Prediction results

The ResNeSt model improved by the ResNet network model, the K Nearest Neighbors algorithm combined with the Coyote optimization algorithm, the hybrid multivariate linear regression (Logistic Regression), the Support Vector Machines (SVMs) combined with the Seagull optimization algorithm, LightGBM, and TabTransformer are standard classification prediction algorithms. This section compares the prediction results of the prediction model proposed in this article with the model built with the above algorithms as the basic algorithm on the sample data set introduced in this article and uses the accuracy and standard deviation of the accuracy, precision, and standard deviation of the precision, the recall and standard deviation of the recall, the specificity and AUC values ​​to statistically analyze the experimental results, as shown in Table 7.

**Table 7:** Comparison of experimental results of Anston model and other algorithms on public datasets.

Basic algorithm	Accuracy and standard deviation	Precision and standard deviation	Recall and standard deviation	Specificity	AUC
ResNeSt^61^	0.782 ± 0.0081	0.833 ± 0.0085	0.847 ± 0.0086	0.852	0.863
COA-KNN^62^	0.774 ± 0.0083	0.818 ± 0.0086	0.823 ± 0.0085	0.833	0.784
MLR-CWLS^63^	0.766 ± 0.0087	0.797 ± 0.0088	0.785 ± 0.0087	0.778	0.819
SOA-SVM^64^	0.758 ± 0.0091	0.775 ± 0.0089	0.767 ± 0.0088	0.747	0.837
LightGBM^48^	0.919 ± 0.0078	0.852 ± 0.0082	0.862 ± 0.0084	0.889	0.873
TabTransformer^46^	0.932 ± 0.0075	0.916 ± 0.0081	0.891 ± 0.0082	0.925	0.926
Anston	0.953 ± 0.0069	0.927 ± 0.0077	0.916 ± 0.0081	0.932	0.935

It can be concluded from Table 7 that the accuracy, precision, and recall of the models constructed by ResNeSt, COA-KNN, MLR-CWLS, and SOA-SVM are all lower than 85%, and the specificity and AUC are lower than 90%. The accuracy of the model constructed by the algorithm designed in this paper reaches 95%, the precision reaches 92%, the recall reaches 91%, the specificity reaches 93%, and the AUC reaches 93%. The comparison of experimental results shows that the accuracy, precision, recall, specificity, and AUC of the model designed in this paper are the highest. To further verify the performance of the model proposed in this paper, this paper uses the nursing home subject data set to test the prediction model, and the test results are shown in Table 8.

**Table 8:** Comparison of experimental results of Anston model and other algorithms on subject datasets.

Basic algorithm	Accuracy and standard deviation	Precision and standard deviation	Recall and standard deviation	Specificity	AUC
ResNeSt^61^	0.853 ± 0.0065	0.835 ± 0.0085	0.868 ± 0.0075	0.825	0.789
COA-KNN^62^	0.797 ± 0.0063	0.807 ± 0.0096	0.786 ± 0.0079	0.764	0.813
MLR-CWLS^63^	0.759 ± 0.0069	0.789 ± 0.0088	0.747 ± 0.0076	0.716	0.836
SOA-SVM^64^	0.716 ± 0.0068	0.721 ± 0.0075	0.735 ± 0.0078	0.697	0.861
LightGBM^48^	0.895 ± 0.0061	0.862 ± 0.0077	0.878 ± 0.0073	0.851	0.897
TabTransformer^46^	0.917 ± 0.0057	0.876 ± 0.0074	0.892 ± 0.0069	0.882	0.945
Anston	0.962 ± 0.0055	0.923 ± 0.0072	0.905 ± 0.0067	0.926	0.952

When using the subject data set for testing, this paper also used accuracy and standard deviation of accuracy, precision and standard deviation of precision, recall and standard deviation of recall, specificity, and AUC values ​​to statistically analyze the experimental results. It can be concluded from Table 8 that the accuracy, precision, recall, specificity, and AUC of the model designed in this paper are the highest.

**Fig. 10:**
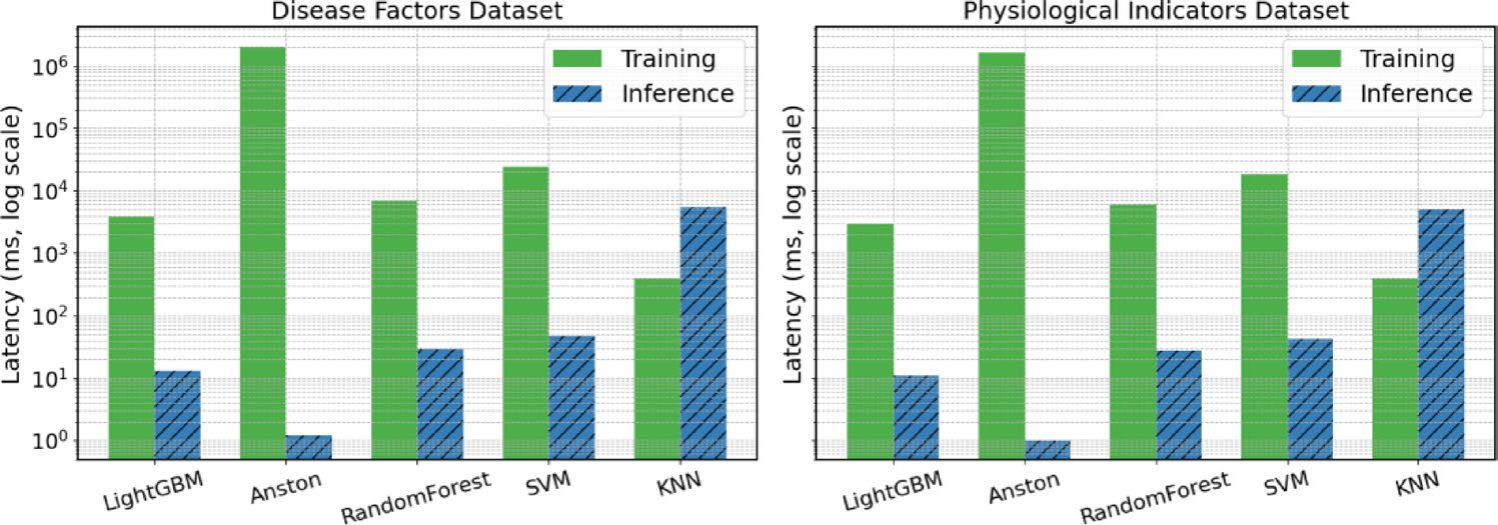
Experiment comparing training time and inference time.

To further understand the performance of the Anston model in terms of execution efficiency, we utilized the public prime data set described in this article to measure the training time and inference time of the Anston model. At the same time, the training and inference times of the LightGBM, TabTransformer, KNN, SVM, and Random Forest models were recorded. The hardware environment features an i7-12700 H CPU, 64 GB of memory, and an RTX 3070 Ti graphics card with 8 GB of video memory. The positive samples of the physiological indicator data set and the disease factor data set described in this article are both 5,000 rows in length, and the negative samples are both 6,000 rows in length. Among them, the Anston model ran 1,000 epochs. The experimental results comparing training time and inference time are shown in Fig. 10.

As shown in Fig. 10, since LightGBM and random forest are tree models, they are swift in training and reasoning in scenarios with small features and thousands of samples. SVM is complex in solving the kernel matrix itself. When the sample size is large, the training and reasoning time will be relatively long. KNN reasoning requires distance calculation for all samples, resulting in a relatively long reasoning time. When training the Anston model, it requires repeated iterations to converge, and the training is the slowest. However, the reasoning is only one forward, so the speed is relatively fast.

**Fig. 11:**
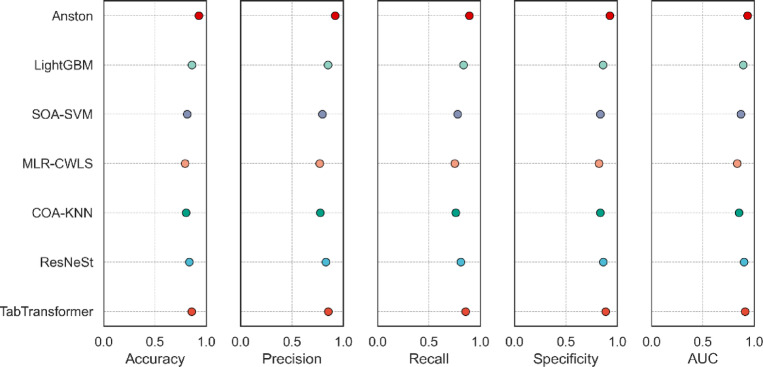
Diabetes prediction comparison experiment.

To promote the research results of this article more widely, we utilized the Pima Indians Diabetes Database to train an Anston model that can predict diabetes risk^65^. The Pima Indians Diabetes Database comprises 768 Pima Indian women aged 21 and above, with 500 being negative and 268 being positive. A comparative experiment was conducted using the diabetes risk prediction model constructed by ResNeSt, COA-KNN, MLR-CWLS, SOA-SVM, LightGBM, and TabTransformer. The evaluation indicators used accuracy, precision, recall, specificity, and AUC values. The comparative experimental results are shown in Fig. 11.

It can be concluded from Fig. 11 that the diabetes risk prediction model trained using the Anston architecture, designed by us, has the best performance in terms of accuracy, precision, recall, specificity, and AUC values compared with the diabetes risk models built using ResNeSt, COA-KNN, MLR-CWLS, SOA-SVM, LightGBM, and TabTransformer.

From the results of the four comparative experiments in this section, it can be concluded that the evaluation index values ​​obtained by the Anston model are better than those of ResNeSt, COA-KNN, MLR-CWLS, SOA-SVM, LightGBM, and TabTransformer. The specific differences between the models are shown in Table 9.

**Table 9:** Differences between models.

Category	Traditional method	Anston	Impact of application scenarios
Category imbalance	ResNeSt/TabTransformer uses BCE by default; LightGBM uses cross entropy + is_unbalance	Focal-LightGBM + Threshold τ = 0.327	Ensure the recall index, which is the primary concern in medical scenarios
Feature weights	Baseline weights are static; SVM/KNN completely relies on distance	Online weight adaptation	Ensure stable performance indicators when encountering feature drift
Structured representation	LightGBM lacks the ability to capture feature interactions; TabTransformer captures interactions but converges slowly	Pre-LN Self-Attn×4 + Cross-Attn×1 + LN-DropPath-GELU FFN	Ensure stable performance indicators when learning implicit relationships
Optimizer/regularizer	AdamW/SGD; ResNeSt needs to readjust lr	Lion + DropPath 0.2: fast convergence and high accuracy	Meet real-time screening and reasoning requirements

As shown in Table 9, Anston adopts a complete link approach that integrates imbalance perception, dynamic weights, efficient attention, and interpretable output and designs a system based on the data characteristics, real-time requirements, and clinical interpretability of imaginative elderly care scenarios. Therefore, it outperforms ResNeSt, COA-KNN, MLR-CWLS, SOA-SVM, LightGBM, and TabTransformer in various evaluation indicators.

The industrial application contribution of this paper is to innovatively apply the model proposed here to the scenario of disease risk prediction in smart elderly care. In the actual environment of competent elderly care, sample imbalance will cause unstable model performance. To solve this problem, we effectively integrate the loss function used in target detection and the attention mechanism network. The above experimental results show that the Anston model proposed in this paper has the highest accuracy, precision, recall, specificity, F1 score, and AUC. Therefore, the disease risk prediction model based on physiological indicators and disease factors proposed in this paper can effectively support the disease risk prediction of wise elderly care.

### Ablation experiment

**Fig. 12:**
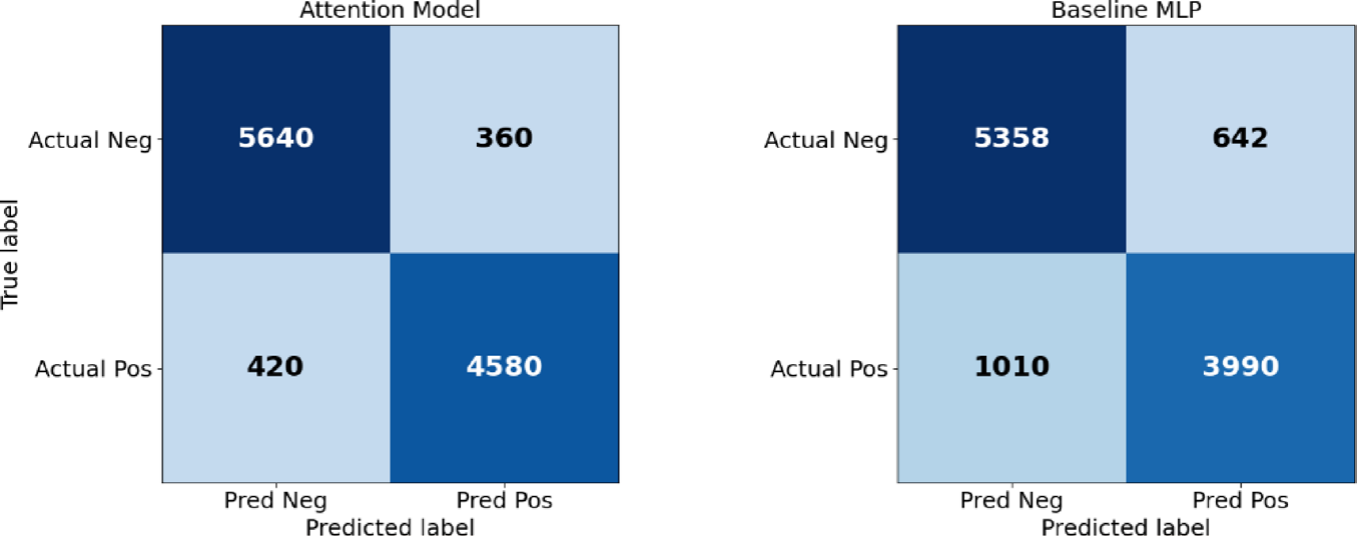
Ablation comparison experiment confusion matrix.

To gain a deeper understanding of the impact of the attention mechanism on the Anston model, we conducted a comparative experiment between the Anston model with and without the attention mechanism. In the ablation comparison experiment, there were 5,000 positive samples and 6,000 negative samples. We used a confusion matrix to analyze the experimental results statistically. See Fig. 12 for details.

**Fig. 13:**
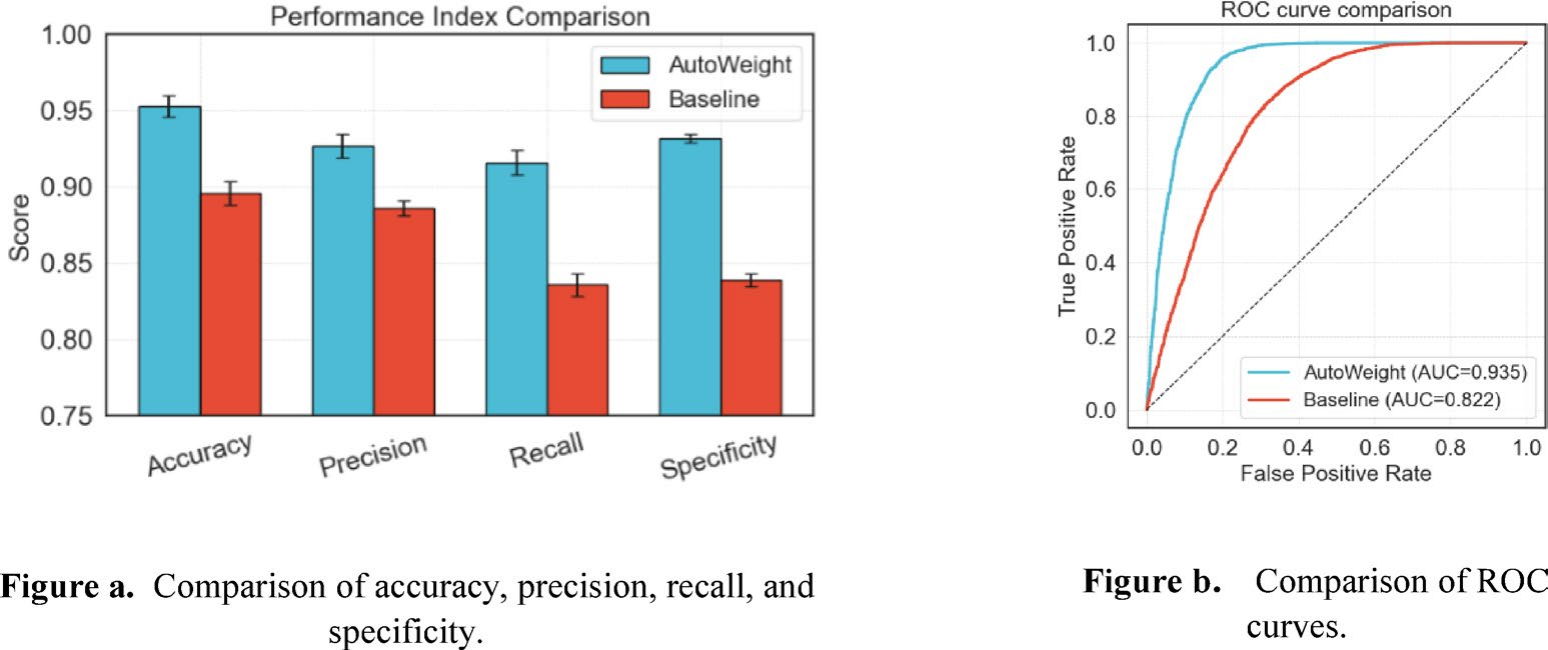
Comparison of automatically updated feature weights.

As shown in Fig. 12, the introduction of the self-attention mechanism enables the model to dynamically adjust weights based on the sample feature values, significantly enhancing the network model’s ability to represent high-order feature interactions. The missed detection rate FN dropped from 1010 to 420, and 590 truly high-risk older adults were missed, which is particularly critical for imaginative elderly care scenarios. The false alarm rate (FP) dropped from 642 to 360, a reduction of 282, and follow-up resources were more focused, thereby reducing the burden on medical care. This not only expands the “correct zone” of true positives and true negatives but also compresses the “error zone” of false alarms and missed alarms so that all key evaluation indicators surpass the model without the introduction of the attention mechanism. To compare the impact of using automatic update feature weights versus not using them on model performance, we conducted a comparative experiment on the Anston model. The results of the comparative experiment are shown in Fig. 13.

Since the important features of the sample are amplified immediately, the weights of the minor or noise features are compressed, which reduces the mismatch caused by overfitting and “concept drift.” Therefore, from Fig. 13(a), we can see that after the Anston model utilizes the automatic update feature weight function, the accuracy, precision, recall rate, and specificity are all improved to some extent. From Fig. 13(b), it can be seen that after the Anston model utilizes the automatic update feature weight function, the AUC value also improves accordingly.

### Model optimization

**Fig. 14:**
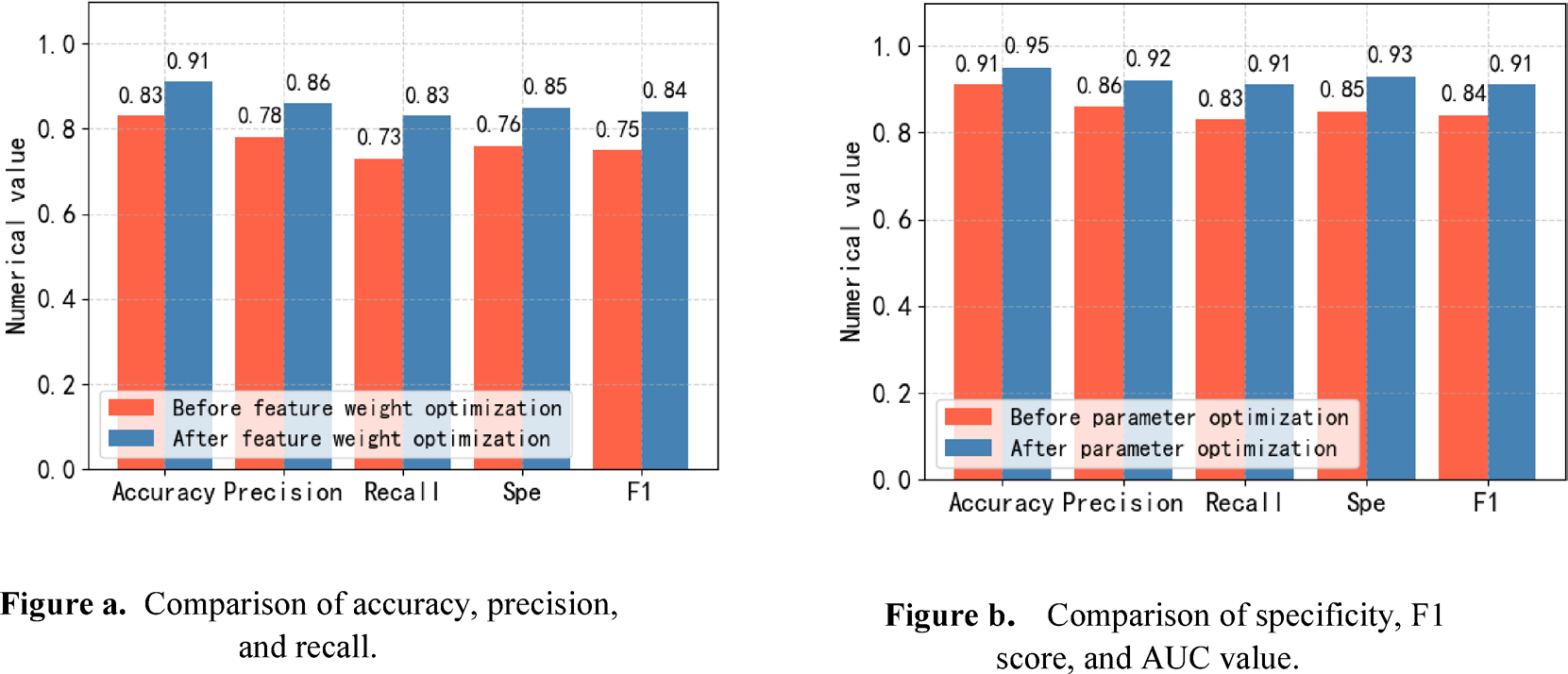
Comparison before and after model optimization. **(a)**: Comparison of prediction results before and after adjustment of feature weights. **(b)**: Comparison of prediction results before and after parameter adjustment.

The model optimization work in this section mainly consists of sample feature weight optimization and model parameter optimization. When optimizing the sample feature weights, use the expression method in the previous section to conduct an in-depth analysis of the influence of each feature and finally obtain the weight of the feature-independent variables. In adjusting the feature weight of the sample data, the feature weight automatic update method is used to adjust the feature weight. The parameters of the prediction model of the feature weight update method consist of model structure parameters, training accuracy parameters, and overfitting parameters. When optimizing the prediction model parameters of the feature weight update method, the GridSearchCV grid search method is used to find and extract the best parameters. When conducting comparative experiments, five evaluation indicators, including accuracy, precision, recall, specificity, and F1 score, were used to compare the prediction results of the prediction models trained before and after the feature weight update. The comparative experimental results are shown in Fig. 14(a).

When optimizing the parameters of the designed attention mechanism network model for structured data classification, the spider wasp optimization algorithm proposed concerning the hunting, nesting, and mating behaviors of female spider bees was used to optimize the parameters. When conducting comparative experiments, five evaluation indicators, including accuracy, precision, recall, specificity, and F1 score, were used to compare the prediction results of the prediction models trained before and after parameter adjustment. The details are shown in Fig. 14(b).

From Fig. 14(a), we can see that the accuracy, precision, recall, specificity, and F1 score of the prediction model after feature weight adjustment are improved compared to the prediction model before feature weight adjustment. From Fig. 14(b), it can be observed that the prediction model after parameter adjustment has a particular improvement in the five indicators of prediction accuracy, precision, recall, specificity, and F1 score compared with the prediction model before parameter adjustment. This phenomenon occurs mainly because, in the prediction model, the default values of parameters have shallow control over the model’s fitting ability, the model’s prevention of over-fitting, and the accuracy of model training. After adjusting the relevant parameters, the values of each evaluation index of the model have been greatly improved.

**Fig. 15:**
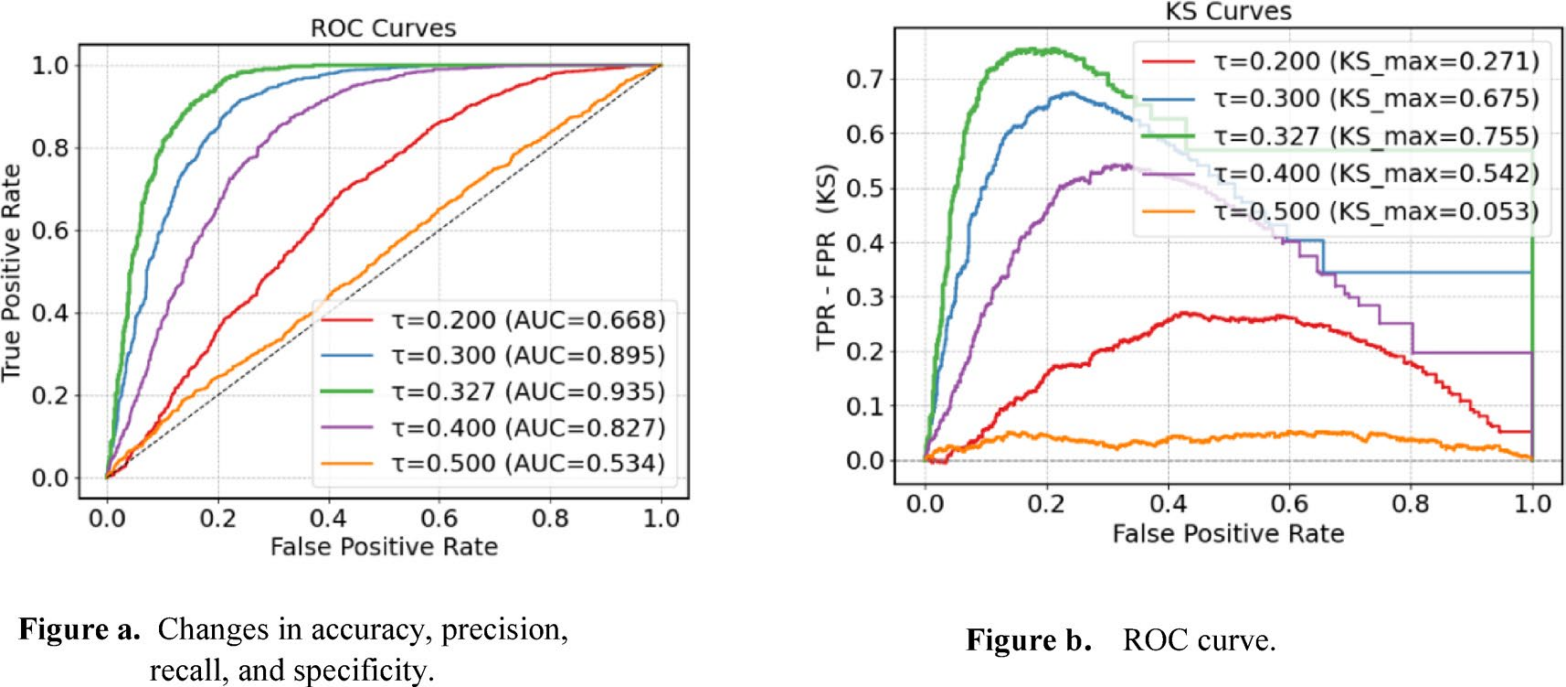
Changes in the classification benchmark threshold during adjustment.

In the research process of this article, in order to solve the problem of unstable prediction model performance caused by the imbalance of the number of positive and negative samples, and the imbalance of the number of easy-to-classify samples and difficult-to-classify samples, we not only integrated the Focal loss function in the target detection network into the integrated learning algorithm LightGBM, but also redefined the classification benchmark threshold in the LightGBM algorithm. During the model optimization process, we selected 5 classification benchmark thresholds (0.2, 0.3, 0.327, 0.4, 0.5), and used ROC curves and KS curves to statistically analyze the performance of the selected 5 classification benchmark thresholds. See Fig. 15 for details.

Figure 15 presents a comparative experiment using a publicly available dataset. It can be seen from Figs. 15(a) and 15(b) that the model’s performance has improved after the classification benchmark threshold was redefined. In the research process of intelligent elderly care disease risk prediction, when there are 1,000 positive samples and 1,200 negative samples, the default threshold of 0.50 will result in a large number of actual positive examples being missed. Shifting the decision threshold to 0.327 can significantly reduce missed detections. The experimental results show that the best model performance is achieved when the threshold is redefined to 0.327.

This section comprehensively explains the Anston model proposed in this paper from the perspectives of datasets, evaluation metrics, and comparative experimental results. The experimental results demonstrate that this model can provide strong technical support for stroke risk prediction in innovative elderly care applications.

## Conclusion

This paper proposes a disease risk prediction model using stroke as an example. To improve the model’s generalization, a method for automatically updating feature weights is designed. To further enhance the model’s stability and predictive performance, a multilayer perceptron neural network and an attention network model for structured data classification are designed. To adapt to the application scenario of disease risk prediction based on physiological indicators and morbidity factors, a disease risk prediction model is proposed that integrates the data augmentation method designed in this paper, the automatic feature weight update method, and the attention network model for structured data classification. Because the Anston model proposed in this study relies on a multilayer perceptron and an attention mechanism, it only provides an end-to-end risk stratification paradigm that can be implemented in resource-constrained nursing homes. In future work, we will continue to advance the risk prediction model in the areas of multi-disease expansion, privacy protection, hardware simplification, and fair interpretation, expanding it from a single-point stroke screening to a comprehensive risk prediction hub for chronic diseases.

## Data Availability

The dataset is from https://physionet.org/content/mimiciii/1.4/ and https://www.kaggle.com/datasets/shashwatwork/cerebral-stroke-predictionimbalaced-dataset. The PIMA diabetes dataset is from https://www.kaggle.com/datasets/uciml/pima-indians-diabetes-database.

## Declarations

### Scientific responsibility statement

The authors declare that they are responsible for the article’s scientific content including study design, data collection, analysis and interpretation, writing, some of the main line, or all of the preparation and scientific review of the contents and approval of the final version of the article.
